# Unraveling Online Perspectives and Misinformation Surrounding Urinary Tract Infections: A Thematic Analysis of 1200 Instagram Posts

**DOI:** 10.5152/tud.2025.25033

**Published:** 2025-06-24

**Authors:** Raya Dean, Patrick Juliebø-Jones, Amelia Pietropaolo, Naeem Bhojani, Wissam Kamal, Bhaskar Somani

**Affiliations:** 1 Department of Urology, University Hospital Southampton, Southamptom, The UK; 2 Department of Urology, Haukeland University Hospital, Bergen, Norway; 3 Division of Urology, Centre Hospitalier de L’Université de Montréal, Quebec, Canada; 4 Urology Unit, King Fahd General Hospital, Jeddah, Saudi Arabia

**Keywords:** Infection, social media, SoMe, urinary tract, urology

## Abstract

**Objective:**

Urinary tract infections are burdensome for patients. Social media is increasingly used as a platform for patients and public to seek and share support. The study aimed to evaluate what patients encounter when they turn to Instagram for urinary tract infection–related support and advice.

**Methods:**

The first 200 posts appearing on the “top posts” section of Instagram for 6 key hashtags (#UTI, #UTIs, #urinarytractinfection, #urinarytractinfections, #bladderinfection, #bladderinfections) were selected. Thematic analysis (TA) was used to identify themes present in the Instagram captions.

**Results:**

Across 1200 posts analyzed, 5 main themes were identified. 1) “We can help…,” this was largely commercial advertising with the promotion of healthcare clinics. 2) “I’m suffering,” which contained first-person narratives about an unpleasant experience with a disease or treatment as well as frustration at health services. 3) “Warning signs,” posts describing signs or symptoms that the creator claims indicate poor health. 4) “Remedies,” these posts detailed therapies to try, often herbal. 5) “Avoid and change,” which covered triggers to avoid symptom flare ups.

**Conclusion:**

There is a large amount of content on social media related to UTIs. Urologists should be aware that patients may have sought out advice using these platforms and may therefore have received misinformation and products that have been advertised but lack scientific evidence.

## Introduction

Urinary tract infections (UTIs) are one of the most common bacterial infections presenting to primary care, affecting up to 60% of adult women in their lifetime.^1^ They are more common in women than men due to the short length of the female urethra and its close proximity to the anus and the vulvar vestibule.^2^ Approximately 400 million cases of UTI were estimated worldwide in 2019,^3^ with UTIs ranking as the leading infection requiring an antibiotic prescription after a doctor’s visit in 2020.^4^ The huge burden of UTIs on individuals and healthcare services indicates the wealth of value to be gained from understanding the barriers that may prevent patients from adhering to medical advice when treating and preventing UTIs.

The use of social media (SoMe) in healthcare is increasing, garnering interest from a multitude of health disciplines as it exhibits usefulness from large-scale public health observation^5^ to smaller-scale provision of health information and resources.^6^ SoMe platforms are frequently used by patients to share their urological health experiences,^7^ which can provide urologists with insight into patients’ experiences with conditions such as UTIs. Existing research has shown that the internet can act as an accessible source of social support that improves patient quality of life,^8^ however also offers an opportunity for misinformation to spread quickly among patients, including medical information that lacks scientific backing.^9^ Launched in 2010, Instagram is one of the most popular SoMe platforms globally, with an estimated 500 million daily users,^10^ the majority of whom are female.^11^

This study aimed to provide healthcare professionals with an understanding of what patients often encounter when they turn to Instagram for UTI-related support and advice.

## Material and Methods

In this study, Instagram post captions constituted the qualitative data that was collected. Ethical approval was granted by the University of Southampton Ethics Committee in August 2023 with the approval number 86364.A1. Data was gathered between September and October 29, 2023, using an anonymous Instagram account created specifically for this study. All data included in the study were sourced from publicly accessible Instagram accounts, in compliance with Instagram’s terms of service and ethical standards for social media research. No private, password-protected, or identifiable personal data were accessed or recorded. To protect user anonymity, no usernames, profile information, or direct quotes that could reveal user identity were included in the publication or analysis. Additionally, URLs were stored only for internal traceability and were not shared or published. Ethical guidelines were followed for internet-mediated research, including those provided by the British Psychological Society and Association of Internet Researchers.

The first 200 posts appearing on the “top posts” section of Instagram for 6 key hashtags (#UTI, #UTIs, #urinarytractinfection, #urinarytractinfections, #bladderinfection, #bladderinfections) were selected, amounting to a total of 1200 posts. Posts were only selected if the caption was written in English, posted within the last 10 years, related to human health, and posted by a public account. The posts were identified via 5 themes based on the recurring phrases or captions into – Promotion of services of products, Symptoms of UTI, Treatment for UTI, Advice for UTI prevention and Health conditions related to UTI. Inter-coder reliability was not assessed, consistent with the principles of reflexive thematic analysis. A reflexivity diary was used, in which team members recorded their assumptions, positionality, and reflections throughout the coding process. Regular analytic meetings were held to challenge interpretations and ensure consistency across the team.

The URLs of each of the 1200 Instagram posts selected were manually copied into an Excel spreadsheet, allowing traceability of the posts so that captions could be collected at a later date. Repeat posts were identified by using conditional formatting on Excel, which highlighted duplicate URLs so that they could be removed. After repeat posts had been removed, Instagram captions and hashtags were copied into an Excel document and then uploaded to NVivo R1. A word frequency query was performed in NVivo R1 to identify the most frequently occurring hashtags in the dataset.

Thematic analysis (TA) was used to identify themes present in the Instagram captions. In this study, TA was carried out with a semantic focus and an essentialist outlook, assuming the possibility of an observable reality in the data. Six phases of analysis were carried out by the authors, in accordance with Braun and Clarke’s approach to reflexive TA.^12^ Familiarisation with the data was achieved through repeated annotation of the captions. Captions were coded in NVivo R1 to identify simple observations in the data. Initial themes were then brainstormed based on these codes, before being developed and refined several times. Reflexivity was achieved through the use of a reflexivity diary. Once reflexive TA was complete, the captions were re-coded using a codebook of subthemes in an attempt the quantify the data in some way. These simple subthemes were created based on existing familiarity with the dataset.

## Results

### Descriptive analysis

The majority of Instagram posts selected were uploaded in 2023 (78%). The most frequently occurring hashtag was #UTI, which featured in over half of the Instagram posts in this study (Table 1). Hashtags relating to women’s health notably featured in the dataset, the most frequent being #womenshealth (n = 264), #vaginalhealth (n = 200), #vaginalcare (n = 131) and #femalehealth (n = 111). Hashtags relating to vaginal infections, such as #yeastinfection (n = 164), #bacterialvaginosis (n = 124), and #yeastinfections (n = 108), also featured prominently in the dataset. In this study, all Instagram posts mentioning ‘yeast infection’ used this term to indicate vaginal candidiasis.

### Thematic Analysis

**Figure 1.**
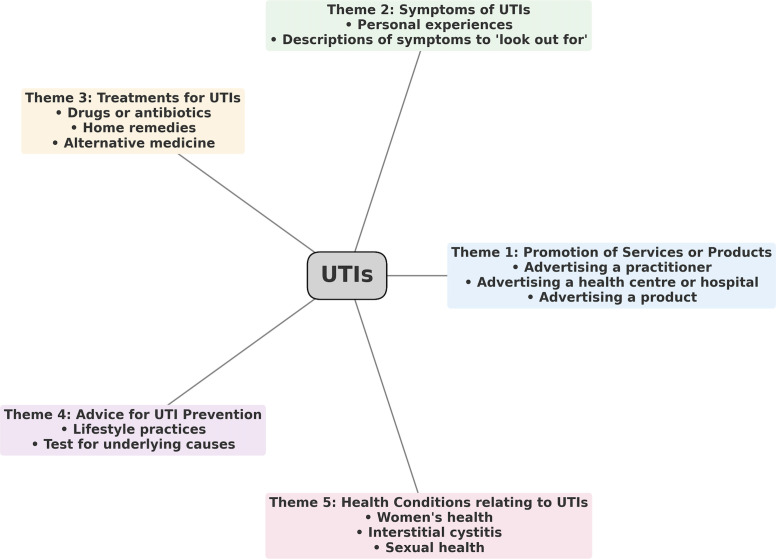
Overview of themes identified.

Reflexive thematic analysis generated 5 main themes: “We can help…,” “I’m in pain,” “Warning signs,” “Remedies,” and “Avoid and change” (Figure 1). The number of posts exhibiting each of these themes (and their subthemes) is reported in Table 2.

Theme 1 – “We can help…”: Posts claiming to be able to improve health or eradicate disease. These posts often urged Instagram users to contact a company or purchase a product or service (which would likely result in financial gain to the creator). This theme was the most prevalent across the dataset (n = 400). The majority of these posts promoted a service (n = 264), which were mostly healthcare clinics. Only a small number of these posts listed medical credentials (n = 30). Several of these posts promoted products that were for sale (n = 130) and a small number included customer testimonials (n = 17).

Theme 2 – “I’m suffering”: Posts containing first-person narratives about an unpleasant experience with a disease or treatment. This was the least prevalent theme in the dataset (n = 89). Just over half of these posts were experiences relating to UTIs (n = 52), while the rest were regarding other conditions such as interstitial cystitis or genitourinary syndrome of menopause. Some creators expressed frustration with healthcare professionals (n = 12). A small number of creators seemed to seek support from peers online (n = 9).

Theme 3 – “Warning signs”: Posts describing signs or symptoms that the creator claims indicate poor health or disease (and therefore should warrant concern). These posts were often portrayed as “red flags” for the Instagram user to look out for. This theme was evident in nearly 20% of Instagram captions in the dataset (n = 227). Less than half of these posts actually described symptoms indicative of UTI (n = 93); the rest seemed to focus on non-related symptoms (indicative of other conditions such as vaginal candidiasis). Some posts encouraged Instagram users to seek medical advice if experiencing symptoms (n = 74), and some encouraged self-diagnosis (n = 26).

Theme 4 – “Remedies”: Posts detailing interventions that the creator claims will improve health or eradicate disease. These posts were often recommended by the creator but had no obvious financial gain. This was the second most prevalent theme in the dataset (n = 316). Most of these remedies were herbal (n = 94), recommending the use of herbs such as Goldenrod or Uva Ursi. Several posts recommended supplements (n = 76), often containing D-mannose, cranberry extract, prebiotics, or probiotics. Less than 10% of these posts recommended medical treatments such as antibiotics (n = 26). A small number of posts encouraged acupuncture or reflexology (n = 3).

Theme 5 – “Avoid and change”: Posts advising Instagram users to avoid certain triggers or change their behaviour in order to improve health or prevent disease. This theme was evident in nearly 15% of Instagram captions in the dataset (n = 164). Many of these posts recommended diet changes and maintaining hydration levels (n = 79). Some of these posts advised specific sexual practices (n = 42), such as urinating after sex. Some of these posts encouraged a change in bathroom habits (n = 35), advising against holding in urine or wiping from back to front.

Most captions contained multiple themes. A small number of captions were deemed not to contain any of the themes outlined above.

## Discussion

Descriptive analysis conducted in this study illustrates that the prevailing context in which UTIs are being discussed on Instagram is one of women’s health, with gynaecological pathologies such as bacterial vaginosis and vaginal candidiasis frequently appearing in hashtags alongside UTIs. This is consistent with current literature that shows that women affected by bacterial vaginosis are more likely to experience UTIs.^13^

Perhaps the most concerning finding in this study is the high prevalence of Instagram users offering unregulated UTI-related products and services for monetary gain. Patients suffering from recurrent UTIs can feel desperate for answers, with previous research showing that many patients will seek alternative strategies to treat their UTIs once they feel that they have exhausted all available options.^14^ Captions that overstate the benefits of these alternative therapies may provide patients with false hope and alter their perceptions of the severity of their UTI. It is important that patients do not ignore key signs and symptoms that indicate serious infection requiring medical attention.

Instagram can provide users who are searching for UTI advice online with some guidance on UTI symptoms to look out for. This may empower patients to accurately self-diagnose their UTIs and consequently seek medical attention. Advice on Instagram regarding the treatment of UTIs seems to favour the recommendation of herbal remedies rather than medical treatments. However, early symptom recognition via social media can be a double-edged sword. On one hand, exposure to posts describing symptoms such as urgency, dysuria, or frequency may empower users to better understand their bodies and seek care more promptly. In this way, social media can act as a low-barrier source of symptom education and help reduce stigma.

However, it is also acknowledged that posts that encourage self-diagnosis without reference to clinical consultation, especially those conflating symptoms of different urogenital conditions may lead individuals to misattribute their symptoms. This could result in inappropriate self-treatment using non-evidence-based remedies, or worse, a delay in accessing medical care when needed. This concern is particularly relevant in posts where the line between personal testimony and health advice is blurred, and where herbal remedies or lifestyle changes are promoted as complete alternatives to medical treatment. Such content may reinforce mistrust in healthcare services, especially among those with unresolved or recurrent symptoms.

Instagram seemed to offer some social support through Instagram users sharing personal experience with UTIs, however this can be difficult to distinguish from promotional testimonials. Exploration of Instagram as a platform for social discussion was limited in this study as only captions were analyzed, not comments. Future research could be conducted to investigate how patient health behaviours change over time following exposure to Instagram-based UTI advice.

### Limitations

The findings of this research were limited to recent Instagram posts, as the ‘top posts’ section of Instagram seemed to overwhelmingly favour posts uploaded in 2023. Analysing Instagram captions only captures a limited fraction of the ‘key messages’ that Instagram users attempt to spread when they upload content. This study did not evaluate any graphics contained in Instagram posts, the accounts and websites that were linked in the post captions, or the discourse taking place in the comments section of each post. Instagram stories, which disappear after 24 hours, were also not included in this research due to practical limitations. It also did not have images, comments, hyperlinks, and Instagram stories, which might limit the interpretability of these findings. These additional components could have provided crucial contextual cues, including visual branding, testimonial emphasis, or emotional tone, which are not captured by caption text alone. However, a sub-sample of top 20 frequently occurring hashtags (Table 1). The scientific accuracy of the posts were also sub-analyzed, and less than half were aligned with the evidence-based practice. This highlights the risk of misinformation and the influence they can have on patient behaviour. Clinicians must be of the content patients are exposed to online and be equipped to address misconceptions during clinical encounters. These findings are from 2023, and Instagram health-related content is highly dynamic and subject to temporal trends, and public discourse on self-care and antibiotic stewardship might influence content. Although “top posts” provide valuable insight into the content most likely encountered by users, they may not reflect the full diversity of patient experience or information quality on the platform.

Although it was not possible to verify personal experience with UTIs or professional urological experience (which can only be self-reported on Instagram), it can be argued that this limitation is not immensely significant. This study aimed to explore the themes that patients are met with when searching Instagram for UTI-related content. Whether Instagram users’ claims are credible or not, these claims can still influence a person’s perceptions and behaviours after they are exposed to them. The themes identified in this study provide an overview of the types of content that frequently appear to Instagram users when they search for UTI-related hashtags on the platform.

### Practical, Actionable Recommendations for Clinicians

Healthcare providers, particularly urologists should proactively engage patients in conversations about where they seek health information online. Given the high volume of UTI-related content on Instagram, often lacking scientific backing or potentially promoting unregulated treatments, it is essential that clinicians address possible misconceptions during consultations. This approach encourages shared decision-making and builds patient trust. Furthermore, clinicians should be prepared to direct patients toward reputable sources of information, such as national guideline repositories or trusted public health websites. Given the overlap between UTI-related content and broader women’s health discourse on social media, clinicians may also consider tailoring their educational materials and social media outreach to include tags and language reflecting this intersection, potentially increasing the visibility of reliable content. Finally, healthcare institutions could explore digital literacy interventions to help patients critically appraise online health claims, thereby reducing the influence of misleading or harmful content.

Healthcare professionals should have an understanding that patients who use Instagram for health advice, especially those who are exasperated from facing unresolved recurrent UTIs, are likely to encounter promotional materials. Patients should be safeguarded from online misinformation and potentially dangerous unlicensed treatments. Considering the reasons behind patient scepticism toward medical treatment, particularly by analysing unfiltered patient perspectives found online, may help to combat misinformation and unnecessary suffering.

Urologists can be aware that UTIs are often discussed on Instagram in the context of female reproductive health, therefore social media campaigns to spread urological information may have a wider reach if they are tagged with pathologies that commonly exist alongside UTIs. It is important that urologists follow professional guidelines when using Instagram to share health advice.

## Figures and Tables

**Table 1.** Top 20 Frequently Occurring Hashtags

Hashtag	Number of Posts
#uti	624
#urinarytractinfection	385
#womenshealth	264
#urinarytractinfections	256
#bladderinfection	246
#utis	231
#bladderinfections	206
#vaginalhealth	200
#yeastinfection	164
#vaginal care	131
#bacterialvaginosis	124
#femalehealth	111
#selfcare	108
#yeastinfections	108
#bladderhealth	99
#reproductivehealth	94
#bladder	93
#health	92
#urology	92
#healthyvagina	87

**Table 2.** Summary of Themes and Subthemes

Theme/Subtheme	Number of Posts
**Theme 1: We can help**	**400**
Promotion of services	264
Promotion of products	130
Medical credentials	30
Customer testimonials	17
**Theme 2: I’m suffering**	**89**
Experiences with UTls	52
Frustration with healthcare professionals	12
Seeking peer support	9
**Theme 3: Warning signs**	**227**
Symptoms of UTI	93
Encourage seeking medical advice	74
Encourage self-diagnosis	26
**Theme 4: Remedies**	**316**
Herbs and botanicals	94
Supplements	76
Medical treatments	26
Acupuncture and reflexology	3
**Theme 5: Avoid and change**	**164**
Diet and hydration	79
Sexual practices	42
Bathroom habits	35

UTI, urinary tract infection.

## Data Availability

The data sets generated during and/or analyzed during the current study are available from the corresponding author on reasonable request.
